# Evolution of Habitat-Dependent Antibiotic Resistance in Pseudomonas aeruginosa

**DOI:** 10.1128/spectrum.00247-22

**Published:** 2022-06-29

**Authors:** Pablo Laborda, José Luis Martínez, Sara Hernando-Amado

**Affiliations:** a Centro Nacional de Biotecnología, CSIC, Madrid, Spain; b Programa de Doctorado en Biociencias Moleculares, Universidad Autónoma de Madrid, Madrid, Spain; Brown University

**Keywords:** experimental evolution, *Pseudomonas aeruginosa*, antibiotic resistance, evolution constraints

## Abstract

Pseudomonas aeruginosa is an opportunistic human pathogen that usually causes difficult-to-treat infections due to its low intrinsic antibiotic susceptibility and outstanding capacity for becoming resistant to antibiotics. In addition, it has a remarkable metabolic versatility, being able to grow in different habitats, from natural niches to different and changing inpatient environments. Study of the environmental conditions that shape genetic and phenotypic changes of P. aeruginosa toward antibiotic resistance supposes a novelty, since experimental evolution assays are usually performed with well-defined antibiotics in regular laboratory growth media. Therefore, in this work we address the extent to which the nutrients’ availability may constrain the evolution of antibiotic resistance. We determined that P. aeruginosa genetic trajectories toward resistance to tobramycin, ceftazidime, and ceftazidime-avibactam are different when evolving in laboratory rich medium, urine, or synthetic sputum. Furthermore, our study, linking genotype with phenotype, showed a clear impact of each analyzed environment on both the fitness and resistance level associated with particular resistance mutations. This indicates that the phenotype associated with specific resistance mutations is variable and dependent on the bacterial metabolic state in each particular habitat. Our results support that the design of evolution-based strategies to tackle P. aeruginosa infections should be based on robust patterns of evolution identified within each particular infection and body location.

**IMPORTANCE** Predicting evolution toward antibiotic resistance (AR) and its associated trade-offs, such as collateral sensitivity, is important to design evolution-based strategies to tackle AR. However, the effect of nutrients' availability on such evolution, particularly those that can be found under *in vivo* infection conditions, has been barely addressed. We analyzed the evolutionary patterns of P. aeruginosa in the presence of antibiotics in different media, including urine and synthetic sputum, whose compositions are similar to the ones in infections, finding that AR evolution differs, depending on growth conditions. Furthermore, the representative mutants isolated under each condition tested render different AR levels and fitness costs, depending on nutrients’ availability, supporting the idea that environmental constraints shape the phenotypes associated with specific AR mutations. Consequently, the selection of AR mutations that render similar phenotypes is environment dependent. The analysis of evolution patterns toward AR requires studying growth conditions mimicking those that bacteria face during *in vivo* evolution.

## INTRODUCTION

Pseudomonas aeruginosa is a nosocomial opportunistic pathogen (1, 2), producing infections in immunocompromised patients and in people with underlying diseases (3–8). This bacterium is able to grow in several body locations, being one of the main causative agents of chronic infections in the lungs of cystic fibrosis (CF) or chronic obstructive pulmonary disease (COPD) patients (3) and also being a major cause of urinary tract infections (UTIs) (9, 10). In addition to its metabolic versatility, P. aeruginosa presents a characteristic low susceptibility to a large variety of antibiotics (11–14), and it has a high capacity to acquire further resistance to antibiotics, something that frequently occurs by the acquisition of mutations in patients under treatment (15–17).

The problem of antibiotic resistance (AR) traditionally has been tackled by introducing novel antibiotics into the market, in a sort of a “Red Queen” strategy. However, this strategy is currently insufficient, and approaches to improve the use of the antibiotics we already have and to reduce the emergence of AR are needed (18). For such conservative interventions focused on the rational design of efficient evolution-based treatments to manage bacterial infections, knowledge of the evolutionary trajectories that bacteria can follow to acquire AR and their associated trade-offs is needed. In fact, adaptive laboratory evolution (ALE) studies have shown that the evolutionary landscapes that bacterial populations submitted to a specific selective pressure follow are limited (19–23), supporting that mutation-driven evolution may be constrained and, hence, may be predictable to some extent. However, reproducibility of such evolution is contingent on several factors, which include resistance level and the impact of each mutation on bacterial fitness, mutation rate, the strength of selection pressure, population bottlenecks, clonal interference, cross-selection, compensatory evolution, collateral sensitivity, and epistasis (24–34), and as we discuss here, it may also be contingent on environmental conditions (i.e., nutrient composition of colonized habitats). That is why the study of constraints of evolution of AR and its associated trade-offs is of relevance in order to rationally design novel strategies to eradicate populations of bacterial pathogens, P. aeruginosa included (35–38).

It has been described in previous studies that the evolution of AR may result in changes of bacterial metabolism and of growth dynamics (39), which might be exploited to tackle AR (40). It is also known that dysregulation of metabolism and infective conditions may affect susceptibility to antibiotics and expression of resistance determinants (41–43). In fact, bacterial metabolism has an effect on the efficacy of certain antibiotics (44–46). Furthermore, it has been determined that changes in metabolism may constrain AR evolution (47). Overall, these studies show that AR and bacterial metabolism are closely interrelated (48). Nevertheless, deeper studies are still required to understand the functional constraints imposed by the environment on the evolution of AR. This is a critical issue since fitness costs associated with resistance acquisition can be metabolically compensated for (49–51), and selection of mutations that compensate for fitness costs depends on the bacterial habitat (38, 52). Therefore, environmental conditions determine the resistant mutants that will be established within a population (47, 52). This has special relevance for metabolically versatile bacteria, such as P. aeruginosa, a pathogen capable of causing infections in distinct body locations, each one presenting different nutrients’ availability and physicochemical composition. In fact, when P. aeruginosa migrates from an environment with limited amount of nutrients to the lungs of a host presenting CF, changes in nutrients’ availability lead to major metabolic modifications that result in increased resistance to oxidative stress and a reduction of cell growth, decreasing effectiveness of antibiotics (53).

In order to know the extent to which the evolutionary trajectories toward AR of P. aeruginosa are affected as a function of the type of infection, we performed ALE experiments mimicking the nutritional compositions present in different body locations. The experiments were performed in the presence of antibiotics commonly used to treat P. aeruginosa infections (tobramycin, ceftazidime and the combination of ceftazidime with the β-lactamase inhibitor avibactam) (54–56) in urine and synthetic sputum, and the results were compared with those obtained in previous ALE assays in the presence of these antibiotics in rich laboratory medium (20, 57). Our results show that both genotypic and phenotypic evolutionary trajectories in the presence of the analyzed antibiotics are contingent on growth conditions.

## RESULTS AND DISCUSSION

### Impact of growth conditions in P. aeruginosa stepwise evolution toward tobramycin, ceftazidime, and ceftazidime-avibactam resistance.

**FIG 1:**
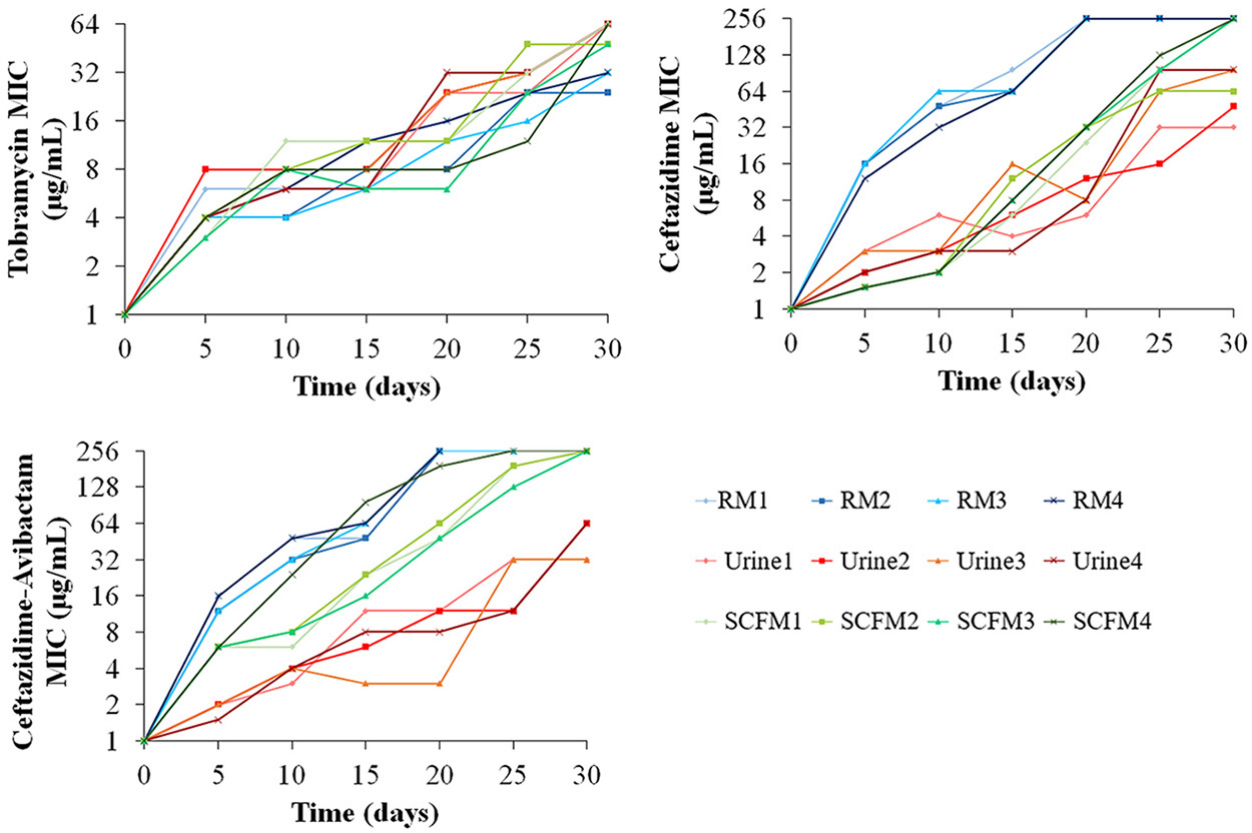
Evolution of P. aeruginosa toward tobramycin, ceftazidime, or ceftazidime-avibactam resistance in rich laboratory medium, urine, or SCFM. MICs for the antibiotic used as selective agent were determined every 5 days in populations that evolved 30 days in the presence of tobramycin, ceftazidime, or ceftazidime-avibactam in rich laboratory medium (RM), urine, or SCFM. Raw data for each evolved replicate are included in Tables S1 and S2.

The aim of the work was to determine the effect of environments similar to those encountered by P. aeruginosa during infections on the evolution of resistance to tobramycin, ceftazidime, and the combination ceftazidime-avibactam. For such a goal, we compared the evolution of AR during 30 days of ALE experiments (four replicates for each environment) in urine and synthetic cystic fibrosis sputum medium (SCFM) with the ones previously obtained in rich laboratory medium (20, 57). The MIC for the antibiotic used as selective agent was measured every 5 days of the ALE, before doubling the concentration of the selective antibiotic. The MICs increased over the evolutionary process in every replicate population, showing stepwise evolutionary trajectories in either urine, SCFM, or rich laboratory medium (Fig. 1; see Tables S1 and S2 in the supplemental material). The evolution of tobramycin resistance showed similar resistance levels after 30 days of evolution in the different media and in the different replicate populations, while in the presence of ceftazidime or its combination with avibactam, differences were observed among populations evolved in rich medium, SCFM, or urine. In particular, the increase of MICs was lower in populations evolved in urine than in rich laboratory medium or SCFM (Fig. 1; Table S2). This indicates that phenotypic evolution of ceftazidime and ceftazidime-avibactam resistance is contingent on environmental conditions.

### Cross-resistance and collateral sensitivity associated with the acquisition of tobramycin, ceftazidime, and ceftazidime-avibactam resistance in P. aeruginosa are dependent on the environment.

**FIG 2:**
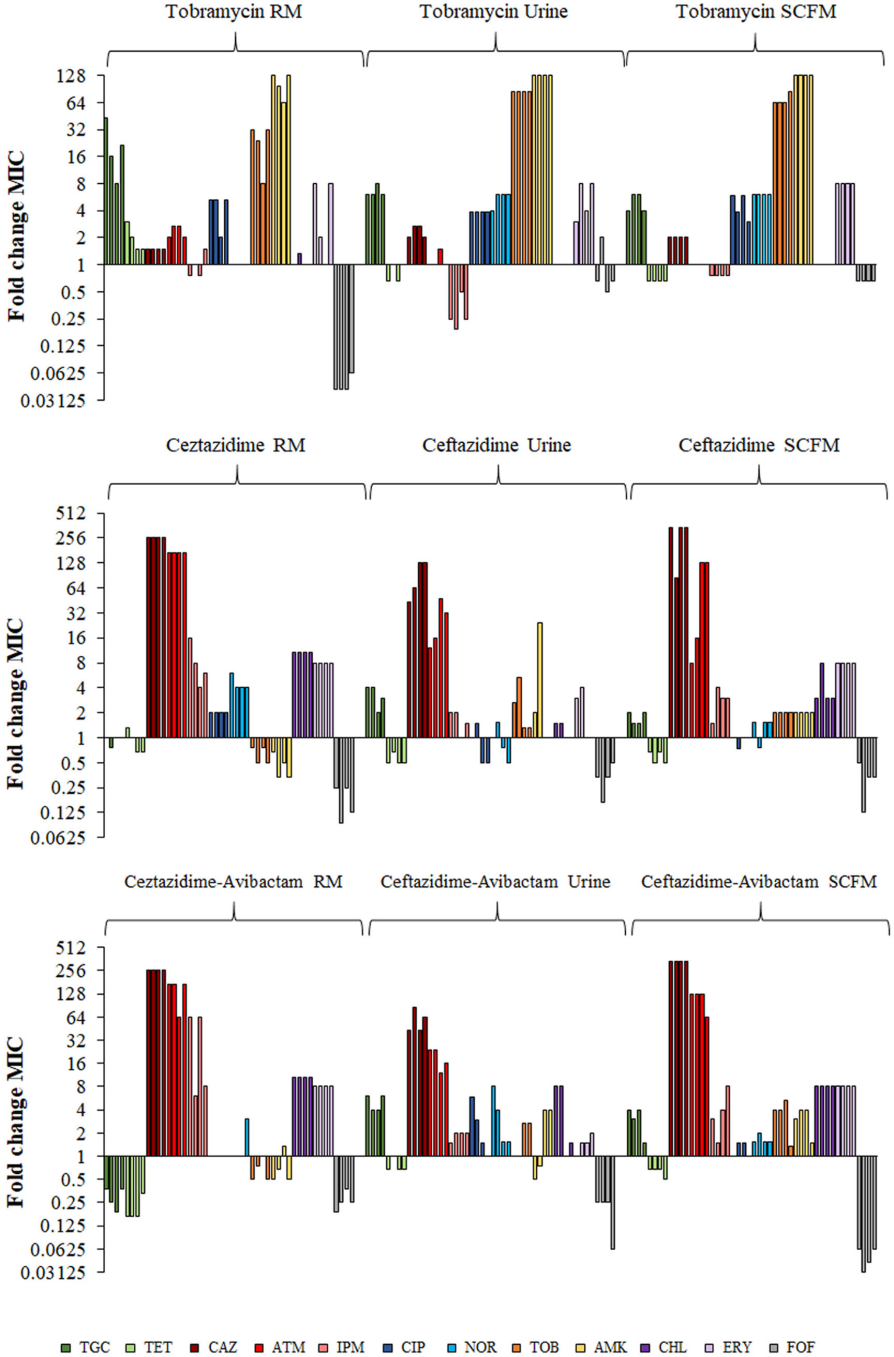
Susceptibility to antibiotics of Pseudomonas aeruginosa populations resulting from evolution in the presence of tobramycin, ceftazidime, and ceftazidime-avibactam in rich laboratory medium (RM), urine, or SCFM. Fold change of MICs of the populations that evolved in the presence of tobramycin (top), ceftazidime (middle), and ceftazidime-avibactam (bottom) in different media was calculated relative to the MIC values of the PA14 parental strain. Fold change MIC values for each antibiotic are represented as bars with the same color and ordered from replicates 1 to 4. Raw data for each evolved replicate population and antibiotic are included in Table S3. TGC, tigecycline; TET, tetracycline; CAZ, ceftazidime; ATM, aztreonam; IPM, imipenem; CIP, ciprofloxacin; NOR, norfloxacin; TOB, tobramycin; AMK, amikacin; CHL, chloramphenicol; ERY, erythromycin; FOF, fosfomycin.

In order to determine the potential cross-resistance and collateral sensitivity patterns associated with the evolution of tobramycin, ceftazidime, and ceftazidime-avibactam resistance in urine, SCFM, or rich laboratory medium, MICs of a set of antibiotics representative of different structural families were determined for the final evolved populations in urine and SCFM and compared with those previously described in rich laboratory medium (20, 57) (Fig. 2; Table S3).

In every replicate population that evolved in the presence of tobramycin, cross-resistance to quinolones, aminoglycosides, or tigecycline was observed, independently of the growth medium used for the experiment (Fig. 2). Nevertheless, collateral sensitivity patterns were dependent on the medium, since a remarkable collateral sensitivity to fosfomycin was only observed in populations that evolved in rich laboratory medium, and an important collateral sensitivity to imipenem was detected only in urine-evolved populations (Fig. 2).

Cross-resistance to β-lactam antibiotics was observed in every ceftazidime- and ceftazidime-avibactam-evolved population. Increased erythromycin and chloramphenicol resistance was also observed in all replicates evolved in rich medium and in SCFM, but was seen in only half of the urine-evolved populations. In addition, populations that evolved in the presence of ceftazidime or ceftazidime-avibactam, in either rich medium, urine, or sputum, presented a robust pattern of collateral sensitivity to fosfomycin. Remarkably, the populations that evolved in the presence of ceftazidime-avibactam in SCFM had a stronger increase in fosfomycin susceptibility than the populations that evolved in other environments. Collateral sensitivity to aminoglycosides was observed only in the populations that evolved in ceftazidime or ceftazidime-avibactam in rich medium, and tigecycline and tetracycline collateral sensitivity was observed only in populations that evolved in the presence of ceftazidime-avibactam in rich medium (Fig. 2).

Overall, these results indicate that the cross-resistance and collateral sensitivity associated with tobramycin, ceftazidime, and ceftazidime-avibactam resistance in P. aeruginosa are contingent on the environment (nutritional composition of growth medium) in which resistance is acquired, although some patterns (i.e., collateral sensitivity to fosfomycin associated with the use of ceftazidime or ceftazidime-avibactam) may be conserved in different environments.

### Genetic basis of resistance to tobramycin, ceftazidime, and ceftazidime-avibactam in different ecosystems.

**FIG 3:**
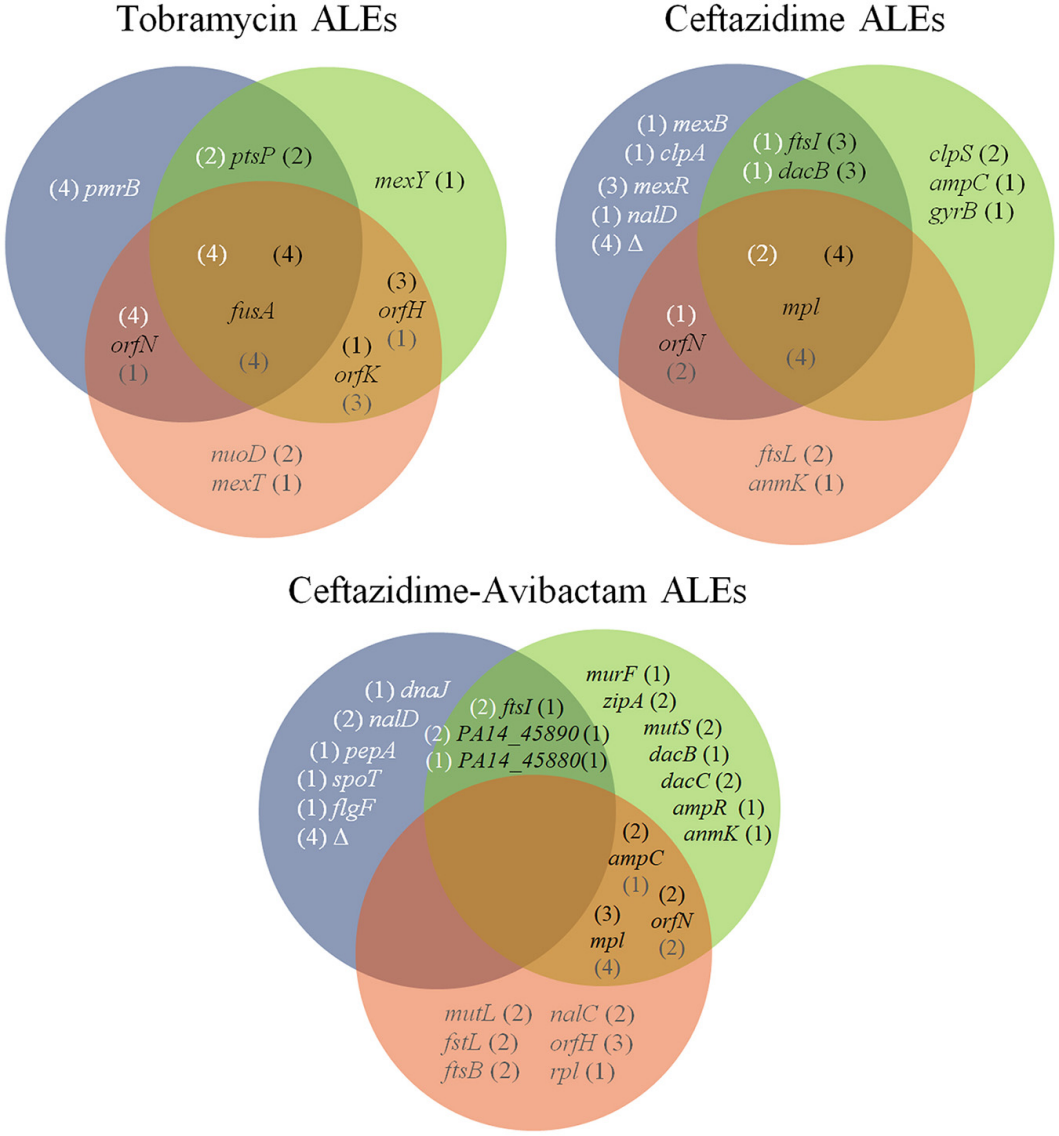
Venn diagram of common and specific genetic modifications in populations evolved in the presence of tobramycin, ceftazidime, and ceftazidime-avibactam in rich laboratory medium, urine, or SCFM. Mutated genes known to be related to AR acquired in rich laboratory medium (20, 57) (blue circles), SCFM (green circles), and urine (red circles) are represented. The numbers in parentheses indicate the number of replicates with mutations in the respective gene in evolutions performed in rich laboratory medium, SCFM, or urine. All detected genetic modifications identified and detailed related information are included in Table S4.

Once we determined that phenotypic AR evolution is contingent on growth conditions, we analyzed the genetic causes of such evolution. In order to identify the genetic modifications responsible for AR in the populations that evolved in urine or SCFM in the presence of tobramycin, ceftazidime, or ceftazidime-avibactam, their genomes, as well as those of control populations grown in the absence of antibiotics, were sequenced after 30 days of evolution. All detected genetic variations are described in Table S4. A search of the mutated genes in the Pseudomonas Genome Database (58) showed that orthologs of all of them are present in different P. aeruginosa isolates, supporting that our findings can be generalized to other strains besides PA14. While, in most cases, the acquired mutations have a negative effect on the activity of the encoded protein—like the ones in the transcription-negative regulators DacB, DacC, MexR, NalC, and NalD, in targets of antibiotics or proteins related to its function, like FtsI, FtsL, FtsB, MurF, AnmK, and Mpl, in proteins that influence the antibiotics’ passage through the membrane, like NuoD or OrfN, or in enzymes involved in general stress responses, like ClpS, ClpA, FusA, and PmrB—the effect is likely positive when the mutation occurs in genes encoding intrinsic resistance determinants, like the subunits of multidrug efflux pumps MexY, MexB, or PA14-45890 or their positive regulators, like AmpR. A Boolean analysis of common and specific genetic modifications acquired in populations evolved in the presence of tobramycin, ceftazidime and ceftazidime-avibactam in rich laboratory medium, urine or SCFM is presented in Fig. 3.

To ascertain if our results reflect *in vivo* evolution toward AR, the identified mutations were searched in the genome of P. aeruginosa clinical isolates using the database BACTOME (59) and by regular bibliographic search. Reinforcing the reliability of our work, several of these genetic changes and most of the mutated genes, although presenting different genetic variations, had been already found in clinical P. aeruginosa isolates, as well as in previous ALE experiments using different P. aeruginosa strains, being related to AR to the analyzed antibiotic (Tables S5 and S6, respectively). Notably, mutations in genes encoding the regulators of the quorum sensing system MvfR (60) and LasR (61) were prevalent in control populations grown in the absence of antibiotics. *lasR* mutants are frequently selected in infected CF patients—more recently it has been described to be selected in a variety of habitats—and this mutation is suggested to be selected as an adaptation to the growing conditions (62–65). However, genetic variations in these genes were absent in ALE assays in the presence of antibiotics (Table S4). This fits with previous results from our laboratory in which mutations in *lasR* were acquired only in control populations. The reason for the absence of mutations in this gene after ALE in the presence of tobramycin or tigecycline was that AR and *lasR* mutations are reciprocally contingent; selection of AR mutations impedes the secondary selection of *lasR-*defective mutants (33). Our results support that not only tigecyline or tobramycin resistance mutations, but also mutations acquired in the presence of ceftazidime or ceftazidime-avibactam, restrict the selection of mutations in *lasR*.

### Effect of the environment on the genetic modifications selected during tobramycin ALEs.

Mutations in *fusA*, encoding an elongation factor (66), were acquired in all replicates of every environment, supporting the importance of mutations within this gene in the acquisition of tobramycin resistance (20), independently of the environment. Indeed, four different genetic variations identified in this work in *fusA* have been described as being associated with acquisition of aminoglycoside resistance, not only in experimental studies (67) but also in clinical strains (59, 68, 69) (Table S5). Mutations in different genes of the *orfKHLN* operon were identified in all replicate populations in every environment. In particular, mutations in *orfN* were acquired in all replicates grown in rich medium, whereas *orfK* and *orfH* mutations were the most prevalent in urine and SCFM, respectively (Table S4). The *orfKHLN* operon encodes the lipopolysaccharide (LPS) O-antigen biosynthesis enzymes (67, 70–72), and it has been proposed that such mutations might reduce drug binding or uptake by alteration of outer membrane (67). The genomic variations detected in *orfN* had been previously described to be involved in tobramycin resistance in experimental studies (33, 67, 73) (Table S5) as well as in collateral sensitivity to fosfomycin in tobramycin-resistant clones obtained after tobramycin ALE in rich medium (36). This is consistent with the fact that only the tobramycin-resistant populations selected in rich medium present a robust collateral sensitivity to fosfomycin (Fig. 2).

Environment-specific mutations were also selected by tobramycin. Mutations in *ptsP*, encoding a phosphoenolpyruvate phosphotransferase, which previously had been related to tobramycin resistance (20, 74), were detected in both rich laboratory medium and in SCFM. More specific was the mutation of *mexY*, encoding a subunit of MexXY efflux pump—an intrinsic aminoglycoside resistance determinant (75)—selected only in SCFM. Interestingly, the His908Leu amino acid change in MexY had been detected previously in tobramycin-resistant clinical isolates (59) (Table S5). Modifications in *nuoD*, which encodes the NADH-quinone oxidoreductase subunit C/D, whose mutations block tobramycin uptake through a disruption of the proton motive force (33, 74), were specifically selected in urine. In addition, a mutation in *mexT*, encoding a regulator of the expression of the genes coding for the MexEF-OprN efflux pump (76) and whose mutations may lead to tobramycin resistance in clinical strains (77), was also selected in this medium. Finally, mutations in *pmrB*, encoding a protein belonging to a two-component regulatory system known to have a role in resistance to polymyxins, fluoroquinolones, β-lactams, aminoglycosides (78), and, specifically, resistance to tobramycin in both experimental (67, 77) and clinical (79) studies, were specifically selected in rich medium.

### Effect of the environment on the genetic modifications selected during ceftazidime ALEs.

The mutations acquired in the presence of ceftazidime were more diverse than the ones selected in the presence of tobramycin, particularly in rich laboratory medium and SCFM. The only gene commonly mutated after ALE in the three different media was *mpl*, which encodes a protein involved in peptidoglycan muropeptide recycling (80). Genetic variations acquired in this gene, leading to Met38fs, Val384Gly, Tyr35Ser, or Val124Gly changes, have also been found in clinical isolates of P. aeruginosa (59, 79) (Table S5). The genetic variation in *orfN* that was selected in the presence of tobramycin or ceftazidime-avibactam (see below) (Fig. 3) was also acquired in ceftazidime, in both urine and rich laboratory medium, supporting the relevant role of this gene in AR evolution of P. aeruginosa. Mutations in *dacB*, encoding a regulator of the expression of the β-lactamase-encoding gene *ampC* (81), and in *ftsI*, which encodes PBP3 (the target of several β-lactam antibiotics) (82), were selected in rich laboratory medium and in SCFM. Those genetic variations leading to a truncated DacB (Gln372*) and to amino acid variations in FtsI (Arg504His and Ala482Val) had been detected previously in clinical isolates (59, 82–84) (Table S5).

Other mutations selected in the presence of ceftazidime were contingent on each specific environment. In SCFM, specific mutations were found in *clpS*, encoding an intracellular protease involved in β-lactam resistance among other physiological processes (85), and in *ampC*, which encodes an intrinsic β-lactamase (86). The Val239Gly amino acid variation in AmpC has been detected in ceftazidime-resistant clinical isolates (59, 79) (Table S5).

Populations evolved in urine presented specific mutations in *anmK*, encoding an enzyme of the peptidoglycan recycling pathway, disruption of which was previously associated with ceftazidime resistance and collateral sensitivity to fosfomycin (87, 88). Interestingly, the genetic variation leading to the Gly232Asp amino acid change has also been detected in ceftazidime-resistant clinical isolates (59) (Table S5). Genetic variations in *ftsL*, encoding a protein needed for FtsI functionality, and leading to a Gly59Asp amino acid change, have been previously described in clinical strains (59) (Table S5). The reduced number of ceftazidime resistance mutations acquired in urine may be responsible for the lower ceftazidime resistance level acquired in these populations than the in other environments analyzed (Fig. 1). Ceftazidime ALE in rich medium led to the acquisition of environment-specific mutations. Some of them may lead to the overexpression of genes encoding MexAB-OprM, an efflux pump that extrudes β-lactams, such as the mutations in *mexR* or *nalD*, encoding its regulators (89, 90). The loss of large chromosomal regions containing *galU* and *mexXY*, previously described to be involved in β-lactam resistance and in intrinsic aminoglycosides resistance, respectively (80), were also specifically selected in populations evolved in rich medium. This explains collateral sensitivity of these populations to aminoglycosides (35, 57), which is absent in urine- and SCFM-evolved populations (Fig. 2).

In agreement with our previous findings showing that fosfomycin collateral sensitivity of rich medium-evolved populations may be associated with mutations in *orfN* (36), we found that fosfomycin collateral sensitivity was conserved in the ceftazidime-evolved populations (Fig. 2).

### Effect of the environment on the genetic modifications selected during ceftazidime-avibactam ALEs.

Evolution in the presence of ceftazidime-avibactam in SCFM and urine led to the selection of genomic variations in *mutS and mutL*, respectively, in some of the evolved populations (Fig. 3). These genes encode components of the mismatch repair system, and their inactivation renders hypermutator phenotypes (91). Since these populations presented a huge number of genetic modifications, only those known to be related to AR are discussed here, in order to simplify such discussion. No common mutations were selected in the three environments. However, in urine and SCFM, common mutations were acquired. Among them, mutations in *mpl*, *ampC*, and *orfN*, also selected in ceftazidime-evolved populations, were selected. Mutations in *mpl* leading to Met38fs and Tyr35Ser were previously detected in P. aeruginosa clinical isolates (59, 79) (Table S5). Evolution in SCFM and rich laboratory medium selected mutations in *ftsI*, which were also selected after ceftazidime ALE, and in *PA14_45890*, encoding an efflux pump involved in P. aeruginosa intrinsic resistance to carbapenems (92, 93) and acquired resistance to ceftazidime-avibactam (57). Finally, growth in SCFM and rich laboratory medium selected mutations in *PA14_45880*, encoding a two-component response regulator. It has been proposed that the two-component system encoded by *PA14_45880-PA14_45870* may regulate the expression of the mentioned *PA14_45890* efflux pump-encoding gene (57).

Among the environment-specific acquired mutations, SCFM ALE selected mutations in *dacB*, *dacC*, *ampR*, *murF*, *zipA*, and *anmK*. Mutations in *dacB*, *dacC*, and *ampR*, which encode regulators of the expression of *ampC* (94), may increase *ampC* expression (95). In addition, the *dacB* genetic variation leading to Trp350Arg was previously detected in ceftazidime-resistant clinical isolates (79) (Table S5). Mutations in *murF*, encoding an enzyme involved in peptidoglycan synthesis, have been described to be involved in Staphylococcus aureus β-lactam resistance (96), and mutations in *zipA*, encoding a cell division protein, are involved in Acinetobacter baumannii β-lactam resistance (97). As mentioned, AnmK participates in the peptidoglycan recycling pathway (98), and its loss of function is associated with β-lactam resistance and collateral sensitivity to fosfomycin (87, 88). However, the mutation in *anmK* was acquired in SCFM and not in urine, as occurred in ceftazidime ALE.

In the case of urine, specific mutations were acquired in *nalC*, encoding a repressor of the expression of the genes encoding the MexAB-OprM efflux pump (99), in *ftsB* and *ftsL*, encoding division proteins from the FtsB/FtsL complex needed for the proper functioning of FtsI (100), and in the transcriptional regulator-encoding gene *rpl*, which produces a transcriptional regulator that regulates the expression of the *dad* operon (101), which controls intracellular d-alanine levels and peptidoglycan synthesis (102).

Apart from genetic modifications in *pepA*, *spoT*, *dnaJ*, and *flgF*, which have been previously related to β-lactam resistance (57), ceftazidime-avibactam rich medium-evolved populations also acquired mutations in *nalD*, encoding a transcriptional regulator of MexAB-OprM efflux pump, and large chromosomal deletions, as those previously mentioned in populations evolved in ceftazidime and rich medium (57), which lead to collateral sensitivity to aminoglycosides (Fig. 2).

Despite the fact that no common genes were mutated in all the environments, it is relevant noting that different mutations might be functionally equivalent and produce similar effects on molecular mechanisms associated with β-lactam resistance. For instance, mutations in *ftsI* or functionally related genes, such as *ftsL* or *ftsB*, were acquired in every medium. Notably, mutations leading to Arg504His in FtsI and to Gly59Asp in FtsL were previously detected in ceftazidime-resistant clinical isolates of P. aeruginosa (59, 82, 83) (Table S5). Furthermore, mutations in genes encoding the regulators of MexAB-OprM efflux pump, such as *nalD* and *nalC*, were selected in urine and in rich medium, respectively, and mutations in the gene encoding the predicted efflux pump, *PA14_45890*, or the genes encoding its likely regulator, *PA14_45880*-*PA14_45870*, were selected in SCFM and rich medium, respectively.

Finally, as it occurred in the ceftazidime evolved populations, fosfomycin collateral sensitivity was acquired independently of the medium used (Fig. 2). As mentioned, this phenotype may be associated with genetic variations in *orfN*, *anmK* (36, 87), or genes related to peptidoglycan synthesis, which is blocked by fosfomycin, such as *murF* and *rpl*.

### Differential fitness cost and levels of resistance are the basis of the differential prevalence of mutations in each environment.

To further understand why specific mutations were acquired in each growth medium, clones from populations evolved in the presence of tobramycin, ceftazidime, or ceftazidime-avibactam were isolated, their mutations were ascertained by Sanger sequencing and those with a representative set of mutations of each ALE were selected for further analysis (Table 1). The isolated representative clones from tobramycin ALE experiments in rich medium, urine, and SCFM were dubbed TobRM, TobU, and TobS, respectively, representative clones of ceftazidime ALE experiments in rich medium, urine, and SCFM were referred to as CazRM, CazU, and CazS, respectively, and representative clones of ceftazidime-avibactam ALE experiments in rich medium, urine, and SCFM were referred to as Caz-AviRM, Caz-AviU, and Caz-AviS, respectively (Table 1).

**TABLE 1:** Genomic variations identified in representative clones of tobramycin and ceftazidime ALEs in urine, SCFM and rich medium

Clone	Gene	Genetic event	Amino acid change^*a*^
TobU	*fusA*	2011A→G	Thr671Ala
	*orfK*	355G→A	Glu119Lys
	*nuoD*	183_184insC	Lys63fs

TobS	*ptsP*	1135dupG	Ala379fs
	*fusA*	2038C→T	Arg680Cys
	*orfH*	286C→T	Arg96*

TobRM	*ptsP*	2156delG	Glu677fs
	*fusA*	1634G→A	Gly545Asp
	*orfN*	148delG	Val50fs
	*pmrB*	853G→C	Val285Leu
			
CazU	*mpl*	353C→G	Thr118Ser
	*ftsL*	176G→A	Gly59Asp

CazS	*mpl*	706A→C	Thr236Pro
	*dacB*	326G→A	Gly109Asp
	*clpS*	248A→C	Gln83Pro
	*ftsI*	1510C→T	Arg504Cys

CazRM	*mpl*	416T→G	Val139Gly
	*dacB*	343C→T	Gly115Ser
	*nalD*	32G→T	Thr11Asn
	*pitA*	367A→C	Thr123Pro
	del299,648 bp	del3200274–3499932	

Caz-AviU	*mpl*	742C→T	Gln248*
	*ftsL*	176G→A	Gly59Asp

Caz-AviS	*anmK*	197G→A	Trp66*
	*mpl*	111delC	Met38fs
	*mpl*	104A→C	Tyr35Ser

Caz-AviRM	*PA14_45890*	1001G→A	Ser334Leu
	*ftsI*	1567C→T	Val523Met
	*ftsI*	1511C→T	Arg504His
	*clpA*	1634A→G	Tyr545Cys
	del220701 bp	del3288650–3509351	

a An asterisk indicates the mutation led to a stop codon.

**FIG 4:**
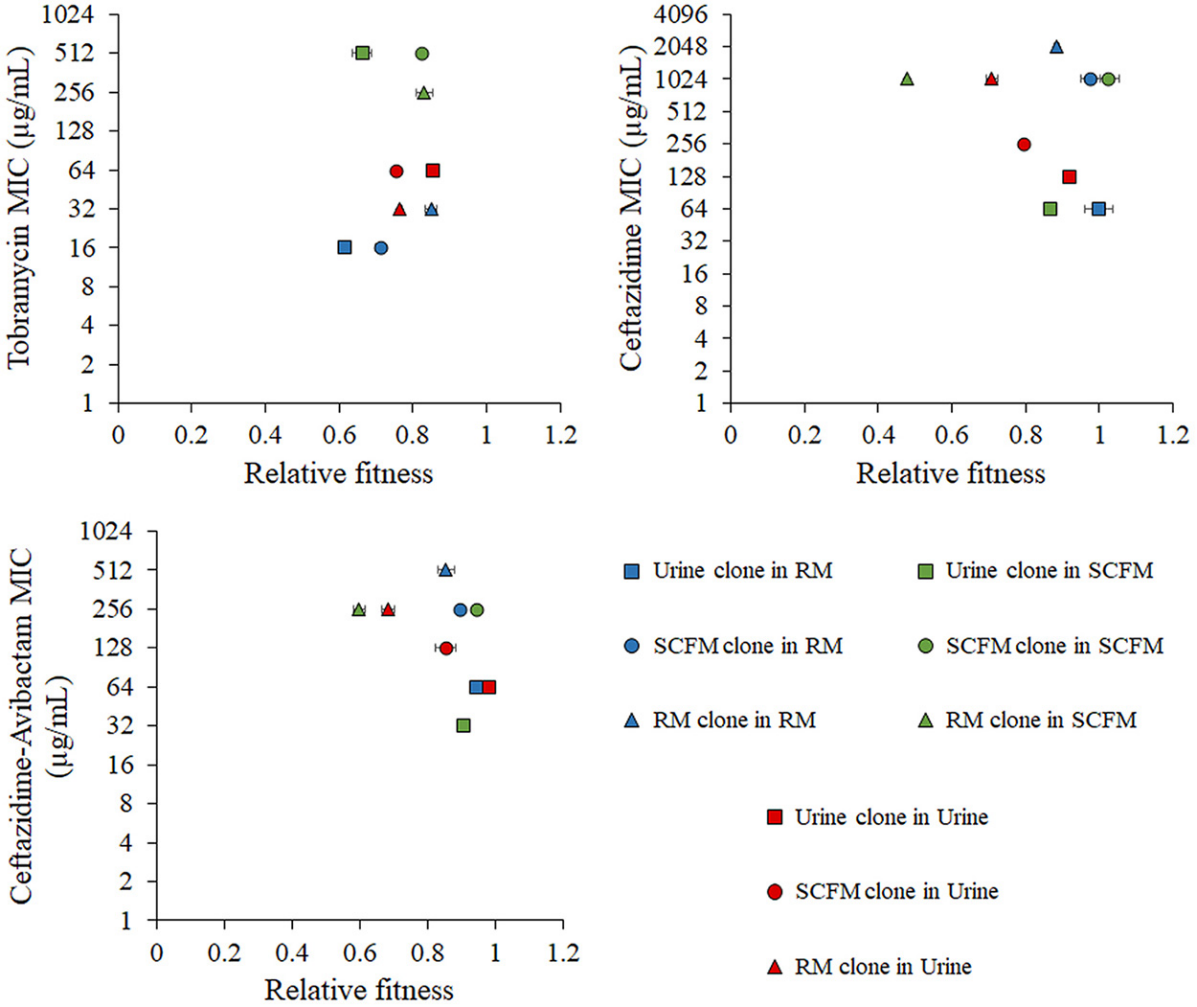
MICs and relative fitness of tobramycin, ceftazidime, and ceftazidime-avibactam ALE representative clones in rich laboratory medium, urine, and SCFM. Growth curves of representative clones of tobramycin, ceftazidime and ceftazidime-avibactam ALEs in urine, SCFM, and rich laboratory medium were recorded in rich laboratory medium, urine, and SCFM. The fitness of each strain was measured as the area under the growth curve. The relative fitness of each clone was calculated with respect to the fitness of PA14 wild-type strain in the same medium. The values shown represent the mean from three replicates. For their part, MICs of tobramycin, ceftazidime and ceftazidime-avibactam clones to the antibiotic in which they were selected were also measured in each medium. The relative fitness of each clone with respect to its MIC values is represented.

The fitness in rich laboratory medium, urine, and SCFM of each representative clone as well as their susceptibility to the respective antibiotic of selection in each medium were measured. Notably, higher MICs do not imply higher fitness costs in the absence of antibiotics (Fig. 4). Differences in MICs were observed for each representative clone in the different media in which they were determined. This finding supports that AR acquisition and the associated trade-offs, as fitness costs or collateral sensitivity, may depend on the place of infection and its nutritional composition, a feature that deserves to be studied in greater depth. Among the three tobramycin representative clones, TobU was that one with highest relative fitness in urine. However, it presented the lowest fitness in rich medium and in SCFM (Fig. 4). This supports that differential fitness costs are based on the selection of the specific set of mutations present in TobU, which was prevalently selected in urine and not selected in SCFM or rich medium. For their part, TobS and TobRM had very similar relative fitness in SCFM (Fig. 4). Nevertheless, the set of mutations acquired in SCFM ALE produces a higher tobramycin resistance level without a relevant fitness cost in SCFM (Fig. 4), therefore being the optimal evolutionary solution for tobramycin selective pressure in SCFM. TobRM had higher tobramycin resistance level and relative fitness than TobS in rich medium (Fig. 4), explaining the selection of the TobRM group of genetic modifications in rich medium (Fig. 4).

Regarding representative clones of ceftazidime ALEs, CazRM presented the highest ceftazidime resistance level in every medium and CazU the lowest (Fig. 4). CazRM, probably due to the loss of a great number of genes (Table 1), presented very low relative fitness in SCFM and urine, but slightly higher fitness in rich medium (Fig. 4). This may indicate that, although the loss of those genomic regions leads to a great level of ceftazidime resistance, its selection is costly, with rich medium the growth medium in which the fitness cost is lower and, hence the medium in which these deletions might be selected (Fig. 4). However, it is worth mentioning that, although these large deletions are not selected *in vitro* in SCFM or urine, clinical P. aeruginosa isolates presenting large deletions are not infrequent (103). CazU was the representative clone with the lowest number of mutations (Table 1) and the lowest level of ceftazidime resistance (Fig. 4). Nevertheless, the ceftazidime resistance level of this clone was higher in urine than in rich medium or SCFM and led to the lowest fitness cost (Fig. 4), explaining the selection of this set of mutations in this medium. Finally, CazS achieved great levels of ceftazidime resistance with a set of mutations that produced the lowest fitness cost in SCFM among the three representative clones (Fig. 4), being the optimal ceftazidime resistance mutational solution in SCFM.

Regarding representative clones of Caz-Avi ALEs, some similarities were observed with that of Caz representative clones. Caz-AviRM presents a large deletion and high resistance levels but very reduced fitness in urine and SCFM, explaining why those deletions are selected only in rich medium. Caz-AviU clones, although presenting the lowest resistance levels, had the lowest fitness cost in urine, being selected in this medium. Finally, Caz-AviS mutations were selected because they produce high levels of resistance and the lowest fitness cost in SCFM.

### Robustness of collateral sensitivity to fosfomycin.

A robust collateral sensitivity to fosfomycin was observed in all the populations that evolved in the presence of ceftazidime or ceftazidime-avibactam in different media (rich medium, SCFM, and urine), but not in the ones that evolved in the presence of tobramycin (Fig. 2). In the last case, collateral sensitivity to fosfomycin was only observed in populations evolved in rich medium. In order to delve into the molecular causes of collateral sensitivity to fosfomycin observed in these populations, the MIC to fosfomycin in all the representative ceftazidime, ceftazidime-avibactam, and tobramycin clones was analyzed. All the ceftazidime and ceftazidime-avibactam representative clones presented increased susceptibility to fosfomycin, but only the tobramycin clone isolated from ALE in rich medium did so, confirming that the ceftazidime and ceftazidime-avibactam ALEs, but not the tobramycin ALE, led to a robust collateral sensitivity to fosfomycin (Table 2).

**TABLE 2:** Fosfomycin MICs of tobramycin, ceftazidime, and ceftazidime-avibactam representative clones at the end of ALE assays in urine, SCFM, or rich laboratory medium and of the PA14 wild-type strain

Strain or clone	Fosfomycin MIC (μg/mL)
Strain	
PA14 (wild type)	32
Clones	
TobRM	8
TobU	48
TobS	32
CazRM	6
CazU	16
CazS	12
Caz-AviRM	6
Caz-AviU	12
Caz-AviS	1.5

**FIG 5:**
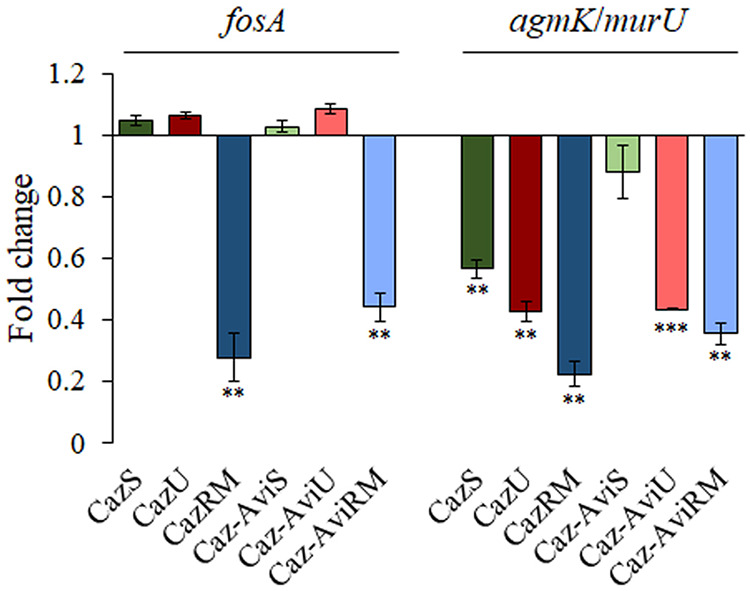
Expression level of genes encoding fosfomycin resistance determinants in the CazS, CazU, CazRM, Caz-AviS, Caz-AviU, and Caz-AviRM clones. Fold changes in expression of CazS, CazU, CazRM, Caz-AviS, Caz-AviU, and Caz-AviRM were calculated relative to the expression of the PA14 wild-type strain and measured by qRT-PCR. Error bars indicate standard deviations from three biological replicates. Statistically significant differences from PA14 were calculated by *t* test for paired samples, assuming equal variances: *, *P* < 0.05; **, *P* < 0.005; ***, *P* < 0.0005.

Collateral sensitivity to fosfomycin in clones from ceftazidime ALEs in rich medium was previously described to be caused by a reduced expression of *fosA*, encoding a fosfomycin-inactivating enzyme, and of genes encoding enzymes from the peptidoglycan recycling pathway (36). Hence, the expression level of such intrinsic fosfomycin resistance determinant-encoding genes was measured in the ceftazidime and ceftazidime-avibactam clones, which presented a robust collateral sensitivity to fosfomycin with respect to the wild type, independent of the medium in which they evolved (rich medium, SCFM, and urine). In agreement with previous information (36), CazRM and Caz-AviRM presented reduced expression levels of both *fosA* and *agmK*/*murU* (encoding the last enzymes of the peptidoglycan recycling pathway) (87) (Fig. 5). For their part, CazS, CazU, and Caz-AviU had reduced expression of *agmK*/*murU* (Fig. 5), which may be related to the genetic variations that these clones acquired in genes encoding proteins associated with synthesis of the cell wall (i.e., in *mpl*, *dacB*, *ftsL*, or *ftsI*). CazRM and Caz-AviRM clones have lower fosfomycin MICs than CazS, CazU, or Caz-AviU clones, possibly because the last ones present impaired expression of the peptidoglycan recycling-encoding genes, but not of *fosA* (Table 2 and Fig. 5). Finally, the Caz-AviS clone acquired a genetic variation leading to a truncated AnmK protein (Table S4), belonging to the peptidoglycan recycling pathway. Although no expression changes in *fosA* were detected in this clone (Fig. 5), it is the clone most susceptible to fosfomycin, confirming that a loss-of-function mutation in *anmK*, whose inactivation has been associated previously with both an increase of ceftazidime resistance and fosfomycin susceptibility (87, 88), has an important impact in this phenotype, as previously described (36).

These results indicate that the robust collateral sensitivity to fosfomycin associated with the acquisition of different ceftazidime resistance mutations in ceftazidime- and ceftazidime-avibactam-evolved populations in SCFM, urine, and rich laboratory medium is caused by reduced activity of the peptidoglycan recycling pathway, either by reduced expression of the genes encoding these enzymes or directly by acquisition of variations in the genes encoding these enzymes.

### Concluding remarks.

Although it has been established that several factors may constrain the evolution of AR (24–34), the extent to which nutritional composition of the colonized environment may modify the evolutionary process in the presence of a specific drug has not been deeply studied, despite the fact that the evolution process largely depends on the habitat where it takes place. In the case of bacterial infections, this can be particularly relevant, since the physicochemical composition, including nutrients’ availability, largely varies in different body locations, a feature that might influence evolution toward AR. Indeed, our results show that the genetic variations acquired by P. aeruginosa in the presence of tobramycin, ceftazidime, or ceftazidime-avibactam are different, depending on whether they are acquired in urine, synthetic sputum, or laboratory rich medium. The reason behind this may be that different mutational patterns lead to different levels of resistance and fitness depending on the composition of growing medium. This indicates that fitness costs associated with specific resistance mutations are not just a nonspecific burden that equally occurs in any ecosystem, but rather fitness costs are habitat dependent. In other words, the phenotype associated with specific resistance mutations is not something rigid and immovable, but it is variable and dependent on the metabolic state of bacteria growing in each particular habitat. This means that different resistance mutations could be selected in different infected locations and that, furthermore, the contribution of these mutations to AR is specific to each environment. It is noteworthy that most of the mutations identified during this work have been previously detected, exactly the same or in the same genes, in antibiotic-resistant clinical isolates of P. aeruginosa (Tables S5 and S6, respectively), supporting the clinical relevance of our results.

The fact that the habitat may restrict the possible selected mutants in the presence of a specific antibiotic has relevant implications for the design of evolution-based approaches to tackle P. aeruginosa infections. Indeed, collateral sensitivity has been explored as a mean to rationally design therapeutic strategies against bacterial infections, but its exploitation largely depends on the robustness of this phenotype in different genetic backgrounds, such as preexisting antibiotic-resistant mutants (35, 37). However, robustness of collateral sensitivity not only implies phenotypic conservation in bacteria presenting different genetic backgrounds evolved in the presence of the same drug, but also refers to the conservation of the phenotype observed when bacteria acquire resistance in different locations within the infected patient, each one presenting a specific nutritional composition. Importantly, we show that collateral sensitivity to fosfomycin associated with the acquisition of ceftazidime and ceftazidime-avibactam resistance is conserved in urine, synthetic sputum, and rich laboratory medium. It is important to emphasize that the latter is the conventionally used medium to experimentally predict patterns of AR and collateral sensitivity, possibly limiting the translation of evolution experiments in clinical settings. This work supports that the evolution of AR in P. aeruginosa is habitat dependent, and therefore, the design of evolutionary strategies to tackle infections should be based on robust patterns of evolution identified within each particular patient environment.

## MATERIALS AND METHODS

### Media and growth conditions.

Overnight cultures were obtained by growing bacteria in lysogeny broth (LB) (Lennox; Pronadisa) at 37°C and shaking at 250 rpm. For the ALE (see below), urine and synthetic cystic fibrosis sputum medium (SCFM) were used. The urine used in this work was obtained by pooling urine samples from four healthy volunteers who had not received antibiotic treatment during the previous year. Urine was then filtered through 0.2-μm-pore-size filters (Whatman) and stored at −20°C until use.

Fresh SCFM was prepared each day for the ALE as described in reference 104, where the concentrations of the components of SCFM were based on the average concentrations of CF sputum samples.

### Antibiotic susceptibility assays.

The concentration of antibiotic (ceftazidime, ceftazidime-avibactam, or tobramycin) used for selection in the ALEs was that one that hinders the growth of P. aeruginosa PA14 parental strain. It was determined in glass tubes in each different medium used for the ALEs at 37°C and 250 rpm.

Susceptibility of evolved populations to different drugs, namely, tigecycline, tetracycline, aztreonam, ceftazidime, imipenem, ciprofloxacin, norfloxacin, tobramycin, amikacin, chloramphenicol, erythromycin, and fosfomycin, was determined by MIC test strips (Liofilchem) in Mueller-Hinton agar (MHA) (Pronadisa) at 37°C following the supplier’s instructions.

MICs of representative clones to ceftazidime or tobramycin in MHA, urine, or SCFM were determined by the broth microdilution method. For that, 96-well plates with round bottoms (Thermo Scientific Nunc) were used. Bacteria were inoculated at an initial optical density at 600 nm (OD_600_) of 0.01, and the concentration of antibiotic in which there was no bacterial growth was determined after incubation at 37°C without shaking for 48 h.

### Adaptive laboratory evolution experiments.

ALE assays were performed as previously described (20, 57), but using SCFM and urine as growth media. Thirty-two bacterial populations of P. aeruginosa PA14 were grown in parallel in urine or SCFM at 37°C with shaking at 250 rpm for 30 days. Four replicates were grown in the presence of either tobramycin, ceftazidime, or the combination ceftazidime-avibactam in SCFM or urine. In addition, eight controls were grown without antibiotic for each medium (4 populations in SCFM and 4 populations in urine). Initial concentrations of antibiotic were determined as previously specified, with 0.5 μg/mL of ceftazidime and 0.5 μg/mL of tobramycin in urine, whereas 3 μg/mL and 2.5 μg/mL of ceftazidime and tobramycin, respectively, were used in SCFM. The avibactam concentration was constantly maintained at 4 μg/mL, as used in clinical tests (105). Each day, the cultures were diluted 1/250 in fresh medium. Every 5 days, the antibiotic concentration was doubled up to 32 times at 30 days. Samples were preserved at −80°C for further research, and MICs for the antibiotic of selection were determined.

### Whole-genome sequencing and bioinformatics analysis.

Genomic DNA of each of the 30-day-evolved populations was extracted by using the Gnome DNA kit (MP Biomedicals, Santa Ana, CA, USA). The DNA quality check and sequencing were performed by Macrogen. Paired-end libraries (2 × 150 bp) were obtained by using the Truseq DNA PCR-free system and sequenced with an Illumina NovaSeq 6000 instrument. The average number of reads per sample represents a coverage of greater than 300×.

Genome sequence, gene coordinates, and annotations were obtained from GenBank. FASTQC was used to verify Illumina short-read quality (106). The alignment of reads against Pseudomonas aeruginosa genome UCBPP-PA14 (GenBank accession no. NC_008463.1) was performed with RNA-STAR (107). The MarkDuplicates (Picard) function of the Genome Analysis Toolkit was used to detect optical and PCR duplicates (108). For indexing of alignment files in BAM format, SAMtools was used (109). Freebayes was used to detect single nucleotide polymorphisms (SNPs) and small insertions and deletions (indels) (110). The impact of indels and SNPs was evaluated with SnpEff (111), and annotated results were saved in VCF format. By using the SNPer viewer (https://bioinfogp.cnb.csic.es/tools/snper) and the IGV browser, genetic variants were detected (112).

The presence of mutations detected in the genome sequencing analysis (see Table S4 in the supplemental material) in representative clones of the treatments was verified by PCR and by Sanger sequencing using the primers shown in Table S7. DNA fragments were purified with the QiAquick PCR purification kit (Qiagen) and Sanger sequenced at Macrogen.

### Relative fitness determination.

Bacteria were inoculated at initial OD_600_ of 0.01 in each well of 96-well microtiter plates with delta surface (Thermo Scientific Nunc) previously filled with 100 μL of LB, urine, or SCFM. Growth curves were obtained by measuring the OD_600_ every 10 min for 48 h at 37°C in a Spark 10M plate reader (Tecan). Triplicates of each condition were performed. The area under the growth curve was considered an estimation of fitness of each bacterial strain in each medium, and relative fitness was calculated with regard to the area under the growth curve of PA14 wild-type strain in the respective medium.

### RNA extraction and qRT-PCR.

Overnight bacterial cultures of the wild-type strain PA14 and of the representative clones CazS, CazU, CazRM, Caz-AviS, Caz-AviU, and Caz-AviRM were inoculated in 20 mL of LB at an OD_600_ of 0.01 and incubated at 37°C and 250 rpm until the exponential phase of growth was reached (OD_600_ of 0.6). Then, the RNA extraction was done as previously described (113), and cDNA was obtained from 10 μg of RNA using the high-capacity cDNA reverse transcription kit (Applied Biosystems).

Quantitative real-time PCR (qRT-PCR) was performed in an ABI Prism 7300 real-time system (Applied Biosystems). Power SYBR green PCR master mix (Applied Biosystems) and 50 ng of cDNA were used in each reaction, which consisted of a denaturation step (95°C for 10 min) followed by 40 cycles of 95°C for 15 s and 1 min at 60°C for amplification and quantification. Primers amplifying a specific fragment of *fosA* (ACCAGGGCGCCTATCTCGAA; CGCTGCGGTTCTGCTTCCAT), *agmK* (AGCTGAATCGCTGGTTGGAC; AACGGTCGGCAGTCTTCCTG), or the housekeeping gene *rplU* (CGCAGTGATTGTTACCGGTG; AGGCCTGAATGCCGGTGATC) (36) were used at 400 nM. The threshold cycle (2^−ΔΔ^*^CT^*) method (114) was used to analyze differences in the relative amounts of mRNA of three independent biological replicates, each containing three technical replicates.

### Data availability.

Whole-genome sequencing data of this work can be found at NCBI with the accession number PRJNA810193.
