# Supplementary material for: Evolution of Habitat-Dependent Antibiotic Resistance in Pseudomonas aeruginosa
Source: Microbiol Spectr. 2022 Jun 29;10(4):e00247-22. doi: 10.1128/spectrum.00247-22 (PMC9431229; doi:10.1128/spectrum.00247-22)
Supplement: Supplemental file 1 — Tables S1 to S7. Download spectrum.00247-22-s0001.pdf, PDF file, 0.4 MB [file spectrum.00247-22-s0001.pdf]

Supplementary Table 1. Tobramycin MICs (μg/mL) for evolved populations in the presence or absence of increasing inhibitory concentrations of tobramycin in rich laboratory medium (MH), urine or SCFM.

Environment	Replicate	5 days	10 days	15 days	20 days	25 days	30 days
MH Tobramycin	1	6	6	12	16	24	24
	2	4	4	8	8	24	24
	3	4	4	6	12	16	32
	4	4	6	12	16	24	32
Urine Tobramycin	1	4	6	6	24	24	64
	2	8	8	8	24	32	64
	3	4	8	8	24	32	64
	4	4	6	6	32	32	64
SCFM Tobramycin	1	3	12	12	12	32	64
	2	4	8	12	12	48	48
	3	3	8	6	6	24	48
	4	4	8	8	8	12	64
MH without antibiotic	1	1	1	1.5	1	1	1
	2	1	1	1.5	1	1	1.5
	3	1	1.5	1.5	1.5	1	1.5
	4	1.5	1	1.5	1.5	1	1.5
Urine without antibiotic	1	1.5	1.5	1	1.5	1	2
	2	1.5	1.5	1	1.5	0.75	1.5
	3	1	1.5	1	1	1.5	2

	4	1	1	1	1.5	1.5	2
SCFM without antibiotic	1	0.75	0.75	1	0.75	0.75	0.75
	2	0.75	0.75	1	0.75	0.75	1
	3	0.75	1	1	0.75	0.75	1
	4	0.75	1	1	0.75	1	1.5

Supplementary Table 2. Ceftazidime MICs (µg/mL) for evolved populations in the presence or absence of increasing inhibitory concentrations of ceftazidime alone or in combination with 4 µg/mL of avibactam in rich laboratory medium (LB), urine or SCFM.

Environment	Replicate	5 days	10 days	15 days	20 days	25 days	30 days
LB Ceftazdime	1	16	48	96	≥256	≥256	≥256
	2	16	48	64	≥256	≥256	≥256
	3	16	64	64	≥256	≥256	-
	4	12	32	64	≥256	≥256	≥256
Urine Ceftazidime	1	3	6	4	6	32	32
	2	2	3	6	12	16	48
	3	3	3	16	8	64	96
	4	2	3	3	8	96	96
SCFM Ceftazidime	1	1.5	2	6	24	96	≥256
	2	1.5	2	12	32	64	64
	3	1.5	2	8	32	96	≥256
	4	1.5	2	8	32	128	≥256
LB Ceftazidime-Avibactam	1	16	48	48	≥256	≥256	≥256
	2	12	32	48	≥256	-	-
	3	12	32	64	≥256	≥256	-
	4	16	48	64	≥256	-	-
	1	2	3	12	12	32	32
	2	2	4	6	12	12	64

Urine Ceftazidime-Avibactam	3	2	4	3	3	32	32
	4	1.5	4	8	8	12	64
SCFM Ceftazidime-Avibactam	1	6	6	24	48	192	≥256
	2	6	8	24	64	192	≥256
	3	6	8	16	48	128	≥256
	4	6	24	96	192	≥256	≥256
LB without antibiotic	1	0.75	0.75	0.75	0.75	0.75	1
	2	1	0.75	0.75	0.75	0.75	1
	3	1	1	0.75	0.75	0.75	0.75
	4	1	1	0.75	0.75	0.75	0.75
Urine without antibiotic	1	1	1.5	0.75	1.5	1.5	2
	2	1.5	1.5	1	1.5	1	2
	3	1	1.5	1	1.5	1.5	2
	4	1	1	0.75	1	1.5	2
SCFM without antibiotic	1	1	1	0.75	1	1	1
	2	0.75	0.75	0.75	1	1	1
	3	0.75	0.75	0.75	1	1	1
	4	0.75	0.75	0.75	1.5	1.5	1.5

- Populations unable to grow after an increase in the antibiotic concentration.

Supplementary Table 3. MICs (μg/mL) of antibiotics from different structural families for final populations submitted to ALE in the presence or absence of tobramycin, ceftazidime or ceftazidime-avibactam in rich laboratory medium (MH or LB, as indicated), urine or SCFM.

Replicate	TGC	TET	CAZ	ATM	IPM	CIP	NOR	TOB	AK	CHL	ERY	FOF
PA14	4	24	0.75	2	1	0.064	0.25	0.75	2	32	32	48
MH Tobramycin												
1	64	48	1.5	3	0.75	0.5	0.25	32	256	24	256	1
2	24	32	1.5	4	1	0.5	0.25	24	192	32	64	1
3	12	24	1.5	4	0.75	0.19	0.25	8	128	24	32	1
4	32	24	1.5	3	1.5	0.5	0.25	32	256	24	256	1.5
Urine Tobramycin												
1	24	16	1.5	2	0.25	0.25	1	64	≥256	32	96	32
2	24	24	2	2	0.19	0.25	1.5	64	≥256	32	≥256	96
3	32	16	2	3	0.5	0.25	1.5	64	≥256	32	128	32
4	24	24	1.5	2	0.25	0.25	1.5	64	≥256	32	≥256	32
SCFM Tobramycin												

1	16	16	1.5	2	0.75	0.38	1.5	64	≥256	32	≥256	32
2	24	16	1.5	2	0.75	0.25	1.5	48	≥256	32	≥256	32
3	24	16	1.5	2	0.75	0.38	1.5	48	≥256	32	≥256	32
4	16	16	1.5	2	0.75	0.19	1.5	64	≥256	32	≥256	32
LB Ceftazidime												
1	2	32	≥256	≥256	8	0.19	1.5	1	1	≥256	≥256	4
2	1.5	24	≥256	≥256	4	0.19	1	1	0.5	≥256	≥256	1.5
3	2	16	≥256	≥256	2	0.19	1	1	0.75	≥256	≥256	4
4	2	16	≥256	≥256	3	0.19	1	1	0.5	≥256	≥256	2
Urine Ceftazidime												
1	16	12	32	24	2	0.064	0.25	2	4	48	96	16
2	16	16	48	32	2	0.094	0.38	4	48	48	128	8
3	8	12	96	96	1	0.032	0.19	1	2	32	32	16
4	12	12	96	64	1.5	0.032	0.125	1	2	32	32	24
SCFM Ceftazidime												
1	8	16	≥256	16	1.5	0.064	0.38	1.5	4	96	≥256	24

2	6	12	64	32	4	0.047	0.19	1.5	4	≥256	≥256	6
3	6	16	≥256	≥256	3	0.064	0.38	1.5	4	96	≥256	16
4	8	12	≥256	≥256	3	0.064	0.38	1.5	4	96	≥256	16
LB Ceftazidime-Avibactam												
1	0.75	4	≥256	≥256	32	0.094	0.25	1	0.75	≥256	≥256	3
2	0.5	4	≥256	≥256	3	0.094	0.25	1	1	≥256	≥256	4
3	0.38	4	≥256	96	32	0.094	0.25	1	2	≥256	≥256	6
4	0.75	8	≥256	≥256	4	0.094	0.75	1	0.75	≥256	≥256	4
Urine Ceftazidime-Avibactam												
1	24	16	32	48	1.5	0.38	2	0.75	1	≥256	32	12
2	16	24	64	48	2	0.19	1	0.75	1.5	≥256	48	12
3	16	16	32	24	2	0.094	0.38	2	8	32	48	12
4	24	16	64	48	2	0.064	0.38	2	8	48	64	3
SCFM Ceftazidime-Avibactam												

1	16	16	≥256	≥256	3	0.064	0.38	3	6	≥256	≥256	3
2	12	16	≥256	≥256	1.5	0.094	0.5	3	8	≥256	≥256	1.5
3	16	16	≥256	≥256	4	0.094	0.38	4	8	≥256	≥256	2
4	6	12	≥256	128	8	0.064	0.38	1	3	≥256	≥256	3
MH												
1	2	12	1.5	2	1	0.094	0.25	1	4	24	32	32
2	2	8	1	2	1	0.19	0.25	1	2	16	32	32
3	1.5	12	1	2	1	0.094	0.25	1	3	32	32	32
4	2	12	1.5	2	1	0.125	0.25	0.75	3	16	32	32
LB												
1	3	12	1	1.5	0.75	0.094	0.25	1	1.5	24	32	24
2	3	12	1	1.5	0.5	0.094	0.25	1	1.5	24	32	24
3	3	12	1	1	0.75	0.094	0.25	1	1.5	24	32	16
4	3	16	1	1	0.75	0.094	0.38	1	1.5	24	32	12
Urine												
1	16	24	2	4	1	0.094	0.38	2	6	32	32	48

2	16	24	2	3	1.5	0.094	0.38	1.5	6	32	48	32
3	16	24	2	3	0.75	0.094	0.38	2	6	32	48	32
4	16	24	2	3	1	0.094	0.38	2	6	32	48	32
SCFM												
1	3	12	1	3	1	0.047	0.19	0.75	2	32	48	64
2	4	12	1	3	1	0.064	0.38	1	3	32	48	32
3	6	16	1	2	1	0.047	0.19	1	2	32	48	32
4	12	12	1.5	4	1	0.064	0.25	1.5	3	32	32	96

TGC, tigecycline; TET, tetracycline; CAZ, ceftazidime; ATM, aztreonam; IPM, imipenem; CIP, ciprofloxacin; NOR, norfloxacin; TOB, tobramycin; AMK, amikacin; CHL, chloramphenicol; ERY, erythromycin; FOF, fosfomycin.

Supplementary Table 4. Newly genetic events acquired after 30 days of ALE in SCFM, urine or rich laboratory medium in the presence of tobramycin, ceftazidime or ceftazidime-avibactam.

Treatment	Replicate	Gene annotation	Gene	Genetic event	Aminoacid change
LB	1	DNA-directed RNA polymerase subunit β	*rpoB*	1562G>A	Gly521Asp
		Transcriptional regulator MvfR	*myfR*	62T>C	Ile21Thr
	2	Transcriptional regulator MvfR	*myfR*	527A>C	His176Pro
		Transcriptional regulator	*psdR*	58A>C	Thr20Pro
		Elongation factor G	*fusA*	1082G>C	Gly361Ala
		Malic enzyme	*maeB*	407T>C	Asp136Gly
	3	Transcriptional regulator MvfR	*myfR*	73G>A	Ala25Thr
		Transcriptional regulator MvfR	*myfR*	727G>T	Glu243*
	4	Transcriptional regulator MvfR	*myfR*	94C>T	His32Tyr

		Transcriptional regulator MvfR	*myfR*	133G>A	Asp45Asn
		Elongation factor G	*fusA*	1082G>C	Gly361Ala
MH	1	LuxR family transcriptional regulator	*lasR*	667_668delGA	Ser223fs
	2	LuxR family transcriptional regulator	*lasR*	667_668delGA	Ser223fs
		DNA-directed RNA polymerase subunit β	*rpoB*	1562G>A	Gly521Asp
	3	LuxR family transcriptional regulator	*lasR*	667_668delGA	Ser223fs
	4	LuxR family transcriptional regulator	*lasR*	667_668delGA	Ser223fs
SCFM	1	DNA-directed RNA polymerase subunit alpha	*rpoA*	867G>T	Leu289Phe
	2	LuxR family transcriptional regulator	*lasR*	349delG	Pro117fs
	3	DNA-directed RNA polymerase subunit alpha	*rpoA*	867G>T	Leu289Phe
	4	LuxR family transcriptional regulator	*lasR*	680C>T	Ala227Val
Urine	1	Helix-turn-helix domain-containing protein	*pcaR*	499A>C	Met167Leu

		Formylglycine-generating enzyme family protein	*pvdO*	794A>G	Asn265Ser
		LuxR family transcriptional regulator	*lasR*	349delG	Pro117fs
		Transcriptional regulator FleQ	*fleQ*	617T>C	Ile206Thr
	2	UDP-N-acetyl-D-mannosamine dehydrogenase	*orfH*	1186G>T	Asp396Tyr
		Transcriptional regulator FleQ	*fleQ*	779G>T	Arg260Leu
	3	Transcriptional regulator FleQ	*fleQ*	1092A>T	Glu364Asp
		Transcriptional regulator FleQ	*fleQ*	809T>G	Val270Gly
	4	LuxR family transcriptional regulator	*lasR*	323_336delCCGGCCTGGTGTATinsACAGCCAGGACTAC	AlaGlyLeuVal108AspSerGlnAsp
		Transcriptional regulator FleQ	*fleQ*	905A>G	Asp302Gly
TOB MH	1	Glycosyltransferase family 4 protein	*orfN*	148delG	Val50fs

	Two-component regulator system signal sensor kinase PmrB	*pmrB*	772C>A	Leu28Met
	Elongation factor G	*fusA*	2011A>G	Thr671Ala
2	Glycosyltransferase family 4 protein	*orfN*	148delG	Val50fs
	Two-component regulator system signal sensor kinase PmrB	*pmrB*	22T>G	Ser8Ala
	Elongation factor G	*fusA*	1783G>C	Ala595Pro
	Transcriptional regulator FleQ	*fleQ*	721T>G	Thr241Pro
	Phosphoenolpyruvate--protein phosphotransferase	*ptsP*	2029G>T	Asp720fs
3	Glycosyltransferase family 4 protein	*orfN*	148delG	Val50fs
	Two-component regulator system signal sensor kinase PmrB	*pmrB*	853G>C	Val285Leu
	Elongation factor G	*fusA*	1634G>A	Gly545Asp

		Transcriptional regulator FleQ	*fleQ*	2156delG	Glu677fs
	4	Glycosyltransferase family 4 protein	*orfN*	148dupG	Val50fs
		Two-component regulator system signal sensor kinase PmrB	*pmrB*	110T>C	Leu37Pro
		Elongation factor G	*fusA*	2038C>T	Arg680Cys
TOB SCFM	1	Elongation factor G	*fusA*	2011A>G	Thr671Ala
		UDP-N-acetyl-D-mannosamine dehydrogenase	*orfH*	792delG	His265fs
		Hypothetical protein	*PA14_44650*	32_33insC	Val12fs
		4-(cytidine 5'-diphospho)-2-C-methyl-D-erythritol kinase	*ispE*	724G>A	Gly242Arg
	2	Elongation factor G	*fusA*	2011A>G	Thr671Ala
		1-deoxy-D-xylulose-5-phosphate synthase	*dxs*	1284_1286delGGTinsCGG	Val429Gly
		1-deoxy-D-xylulose-5-phosphate reductoisomerase	*dxr*	1019C>T	Ala340Val

		UDP-N-acetyl-D-mannosamine dehydrogenase	*orfH*	628delA	Met210fs
	3	Phosphoenolpyruvate--protein phosphotransferase	*ptsP*	520C>T	Gln174*
		Elongation factor G	*fusA*	2033A>T	Gln678Leu
		UDP-N-acetylglucosamine 2-epimerase	*orfK*	646C>T	Arg216Cys
		Multidrug efflux RND transporter permease subunit MexY	*mexY*	2723A>T	His908Leu
		Multidrug efflux RND transporter permease subunit MexY	*mexY*	2729T>C	Val910Ala
	4	Phosphoenolpyruvate--protein phosphotransferase	*ptsP*	1135dupG	Ala379fs
		Elongation factor G	*fusA*	2038C>T	Arg680Cys
		UDP-N-acetyl-D-mannosamine dehydrogenase	*orfH*	286C>T	Arg96*
		UDP-N-acetyl-D-mannosamine dehydrogenase	*orfH*	792delG	His265fs
TOB Urine	1	Elongation factor G	*fusA*	2011A>G	Thr671Ala

	UDP-N-acetylglucosamine 2-epimerase	*orfK*	355G>A	Glu119Lys
	Sigma-54-dependent Fis family transcriptional regulator	*pilR*	301T>C	Phe101Leu
2	Elongation factor G	*fusA*	2011A>G	Thr671Ala
	UDP-N-acetylglucosamine 2-epimerase	*orfK*	355G>A	Glu119Lys
	NADH-quinone oxidoreductase subunit C/D	*nuoD*	183_184insC	Lys63fs
3	Elongation factor G	*fusA*	1366A>G	Thr456Ala
	UDP-N-acetyl-D-mannosamine dehydrogenase	*orfH*	68C>A	Ala23Asp
	Glycosyltransferase family 4 protein	*orfN*	148delG	Val50fs
	LysR family transcriptional regulator	*mexT*	833G>T	Arg278Leu
4	Elongation factor G	*fusA*	2011A>G	Thr671Ala
	UDP-N-acetylglucosamine 2-epimerase	*orfK*	355G>A	Glu119Lys

		NADH-quinone oxidoreductase subunit C/D	*nuoD*	183_184insC	Lys63fs
		Malto-oligosyltrehalose synthase	*PA14_36605*	2754C>G	Tyr918*
CAZ LB	1	Multidrug resistance operon repressor MexR	*mexR*	80_81insA	Glu27fs
		RND multidrug efflux transporter MexB	*mexB*	1126C>G	Leu376Val
		Penicillin binding protein 3 FtsI	*ftsI*	1510G>A	Arg504Cys
		ATP-dependent Clp protease	*clpA*	515delA	His172fs
		del 220701 bp	Δ	del *PA14_36910 – PA14_39440*	
	2	UDP-N-acetylmuramate:L-alanyl-gamma-D-glutamyl-meso-diaminopimelate ligase	*mpl*	1305_1313delCGGCGGCT T	Phe438_Gly440del
		UDP-N-acetylmuramate:L-alanyl-gamma-D-glutamyl-meso-diaminopimelate ligase	*mpl*	371T>G	Val124Gly
		Glycosyltransferase family 4 protein	*orfN*	148dupG	Val50fs

	Translation initiation factor IF-2	*infB*	2407C>T	Val803Ile
	Phosphate transporter PitA	*pitA*	133A>C	Thr45Pro
	del 299658 bp	Δ	del *PA14_35950 – PA14_39320*	
3	Multidrug resistance operon repressor MexR	*mexR*	126_128delAAT	Leu43del
	Heat shock protein GrpE	*grpE*	39delG	Glu14fs
	ATP-dependent Clp protease proteolytic subunit	*clpP*	365delC	Gly122fs
	Molecular chaperone DnaK	*dnaK*	1115G>A	Ala372Val
	RND multidrug efflux transporter MexB	*mexB*	2300T>G	Val767Gly
	del 258271 bp	Δ	del *PA14_36050 – PA14_38910*	
4	UDP-N-acetylmuramate:L-alanyl-gamma-D-glutamyl-meso-diaminopimelate ligase	*mpl*	416T>G	Val139Gly

		Phosphate transporter PitA	*pitA*	367A>C	Thr123Pro
		DNA-directed RNA polymerase subunit β	*rpoB*	2867_2878delTGCAACTCGACC	Met956_Gln960delinsLys
		D-alanyl-D-alanine carboxypeptidase/D-alanyl-D-alanine-endopeptidase	*dacB*	343C>T	Gly115Ser
		Multidrug resistance operon repressor NalD	*nalD*	32G>T	Gly115Ser
		del 299658 bp	Δ	del *PA14_35950 – PA14_39320*	
CAZ SCFM	1	Class C β-lactamase PDC-34	*ampC*	742G>A	Gly248Ser
		Class C β-lactamase PDC-34	*ampC*	716T>G	Val239Gly
		UDP-N-acetylmuramate:L-alanyl-gamma-D-glutamyl-meso-diaminopimelate ligase	*mpl*	1151T>G	Val384Gly
		D-alanyl-D-alanine carboxypeptidase/D-alanyl-D-alanine-endopeptidase	*dacB*	1169G>A	Ser390Asn

	Penicillin binding protein 3 FtsI	*ftsI*	1511G>A	Arg504His
	DNA topoisomerase (ATP-hydrolyzing) subunit B	*gyrB*	2230T>G	Leu744Val
2	UDP-N-acetylmuramate:L-alanyl-gamma-D-glutamyl-meso-diaminopimelate ligase	*mpl*	1151T>G	Val384Gly
	D-alanyl-D-alanine carboxypeptidase/D-alanyl-D-alanine-endopeptidase	*dacB*	270C>G	Tyr90*
	UDP-N-acetylmuramate:L-alanyl-gamma-D-glutamyl-meso-diaminopimelate ligase	*mpl*	706A>C	Thr236Pro
3	D-alanyl-D-alanine carboxypeptidase/D-alanyl-D-alanine-endopeptidase	*dacB*	326G>A	Gly109Asp
	ATP-dependent Clp protease adapter ClpS	*clpS*	248A>C	Gln83Pro
	Penicillin binding protein 3 FtsI	*ftsI*	1510C>T	Arg504Cys
4	UDP-N-acetylmuramate:L-alanyl-gamma-D-glutamyl-meso-diaminopimelate ligase	*mpl*	337G>T	Gly113Cys

		D-alanyl-D-alanine carboxypeptidase/D-alanyl-D-alanine-endopeptidase	*dacB*	1114C>T	Gln372*
		ATP-dependent Clp protease adapter ClpS	*clpS*	288delC	Glu97fs
		Penicillin binding protein 3 FtsI	*ftsI*	1445C>T	Ala482Val
		Penicillin binding protein 3 FtsI	*ftsI*	1244C>T	Ala415Val
CAZ Urine	1	Anhydro-N-acetylmuramic acid kinase	*anmK*	695G>A	Gly232Asp
		UDP-N-acetylmuramate:L-alanyl-gamma-D-glutamyl-meso-diaminopimelate ligase	*mpl*	111dupC	Met38fs
		Glycosyltransferase family 4 protein	*orfN*	148dupG	Val50fs
		Pyoverdine export/recycling transporter periplasmic adaptor subunit PvdR	*pvdR*	669G>A	Met223Ile
		Amino-acid N-acetyltransferase	*argA*	1208T>C	Phe403Ser
	2	UDP-N-acetylmuramate:L-alanyl-gamma-D-glutamyl-meso-diaminopimelate ligase	*mpl*	111dupC	Met38fs

		Glycosyltransferase family 4 protein	*orfN*	148dupG	Val50fs
		Glutamate 5-kinase	*proB*	866C>T	Ala289Val
		50S ribosomal protein L27	*rpmA*	10delA	Ala6fs
	3	UDP-N-acetylmuramate:L-alanyl-gamma-D-glutamyl-meso-diaminopimelate ligase	*mpl*	353C>G	Thr118Ser
		Cell division protein FtsL	*ftsL*	176G>A	Gly59Asp
	4	UDP-N-acetylmuramate:L-alanyl-gamma-D-glutamyl-meso-diaminopimelate ligase	*mpl*	353C>G	Thr118Ser
		Cell division protein FtsL	*ftsL*	176G>A	Gly59Asp
CAZ-AVI LB	1	Chaperone protein DnaJ	*dnaJ*	1081G>A	Pro361Ser
		Multidrug efflux RND transporter permease subunit	*PA14_45890*	1001G>A	Ser334Leu
		Penicillin binding protein 3 FtsI	*ftsI*	1567C>T	Val523Met
		Penicillin binding protein 3 FtsI	*ftsI*	1511C>T	Arg504His

	ATP-dependent Clp protease	*clpA*	1634A>G	Tyr545Cys
	del 220701 bp	Δ	del *PA14_36910 – PA14_39440*	
2	Leucyl aminopeptidase	*pepA*	1439C>T	Gly480Asp
	Carboxyl-terminal processing protease	*ctpA*	971G>A	Ser324Asn
	PII uridylyl-transferase	*glnD*	2466_2467insG	Asp823fs
	Flagellar basal body rod protein FlgF	*flgF*	676C>A	Glu226*
	Multidrug resistance operon repressor NalD	*nalD*	32G>T	Gly115Ser
	RND multidrug efflux transporter MexB	*mexB*	1345C>T	Leu449Phe
	del 55009 bp	Δ	del *PA14_37950 – PA14_38580*	
3	Two component response regulator	*PA14_45880*	159C>A	Met53Ile
	Multidrug efflux RND transporter permease subunit	*PA14_45890*	836G>A	Pro279Leu

		Multidrug efflux RND transporter permease subunit	*PA14_45890*	830G>A	Ala277Val
		del 442741 bp	Δ	del *PA14_35970 – PA14_40880*	
	4	Protein-L-isoaspartate O-methyltransferase	*pcm*	530_531insG	Arg180fs
		Guanosine-3',5'-bis(diphosphate) 3'-pyrophosphohydrolase	*spoT*	931T>C	Phe311Leu
		Multidrug resistance operon repressor NalD	*nalD*	32G>T	Gly115Ser
		RND multidrug efflux transporter MexB	*mexB*	1693C>T	Pro565Ser
		Penicillin binding protein 3 FtsI	*ftsI*	851C>T	Arg284Gln
		del 55009 bp	Δ	del *PA14_37950 – PA14_38580*	
CAZ-AVI SCFM	1**	class C β-lactamase PDC-34	*ampC*	716T>C	Val239Ala
		UDP-N-acetylmuramate:L-alanyl-gamma-D-glutamyl-meso-diaminopimelate ligase	*mpl*	111dupC	Met38fs

	D-alanyl-D-alanine carboxypeptidase	*dacC*	688A>G	Thr230Ala
	D-alanyl-D-alanine carboxypeptidase	*dacC*	697G>A	Ala233Thr
	DNA mismatch repair protein MutS	*mutS*	2026A>C	Thr676Pro
	Glycosyltransferase family 4 protein	*orfN*	148delG	Val50fs
	D-alanyl-D-alanine carboxypeptidase/D-alanyl-D-alanine-endopeptidase	*dacB*	1048T>C	Trp350Arg
	Cell division protein ZipA	*zipA*	76C>T	Arg26Trp
	UDP-N-acetylmuramoyl-tripeptide--D-alanyl-D-alanine ligase	*murF*	574A>G	Thr192Ala
	Class C β-lactamase PDC-34	*ampC*	1040A>G	Asn347Ser
2**	Class C β-lactamase PDC-34	*ampC*	716T>C	Val239Ala
	LysR family transcriptional regulator AmpR	*ampR*	275A>G	Tyr92Cys

	UDP-N-acetylmuramate:L-alanyl-gamma-D-glutamyl-meso-diaminopimelate ligase	*mpl*	111dupC	Met38fs
	D-alanyl-D-alanine carboxypeptidase	*dacC*	688A>G	Thr230Ala
	DNA mismatch repair protein MutS	*mutS*	2026A>C	Thr676Pro
	Glycosyltransferase family 4 protein	*orfN*	148delG	Val50fs
	Cell division protein ZipA	*zipA*	76C>T	Arg26Trp
3	Anhydro-N-acetylmuramic acid kinase	*anmK*	197G>A	Trp66*
	UDP-N-acetylmuramate:L-alanyl-gamma-D-glutamyl-meso-diaminopimelate ligase	*mpl*	111delC	Met38fs
	UDP-N-acetylmuramate:L-alanyl-gamma-D-glutamyl-meso-diaminopimelate ligase	*mpl*	104A>C	Tyr35Ser
4	Two component response regulator	*PA14_45880*	1255C>T	Arg419Cys
	Multidrug efflux RND transporter permease subunit	*PA14_45890*	830C>T	Ala277Val

		Multidrug efflux RND transporter permease subunit	*PA14_45890*	1787A>G	Asn596Ser
		Penicillin binding protein 3 FtsI	*ftsI*	1511G>A	Arg504His
CAZ-AVI Urine	1**	UDP-N-acetylmuramate:L-alanyl-gamma-D-glutamyl-meso-diaminopimelate ligase	*mpl*	111dupC	Met38fs
		Efflux system transcriptional repressor NalC	*nalC*	442_443insC	His150fs
		Cell division protein FtsB	*ftsB*	121A>G	Ile41Val
		UDP-N-acetyl-D-mannosamine dehydrogenase	*orfH*	792dupG	His265fs
		DNA mismatch repair endonuclease MutL	*mutL*	1306A>C	Ser436Arg
	2	UDP-N-acetylmuramate:L-alanyl-gamma-D-glutamyl-meso-diaminopimelate ligase	*mpl*	742C>T	Gln248*
		UDP-N-acetyl-D-mannosamine dehydrogenase	*orfH*	561_565delGGCAGinsAGCTT	Ala189Ser
		Glycosyltransferase family 4 protein	*orfN*	148delG	Val50fs

	Cell division protein FtsL	*ftsL*	176G>A	Gly59Asp
3**	Class C β-lactamase PDC-34	*ampC*	1040A>G	Asn347Ser
	UDP-N-acetylmuramate:L-alanyl-gamma-D-glutamyl-meso-diaminopimelate ligase	*mpl*	111dupC	Met38fs
	Efflux system transcriptional repressor NalC	*nalC*	442_443insC	His150fs
	Cell division protein FtsB	*ftsB*	119_121delAGAinsTGG	GlnIle40LeuVal
	UDP-N-acetyl-D-mannosamine dehydrogenase	*orfH*	792dupG	His265fs
	DNA mismatch repair endonuclease MutL	*mutL*	1306A>C	Ser436Arg
4	UDP-N-acetylmuramate:L-alanyl-gamma-D-glutamyl-meso-diaminopimelate ligase	*mpl*	742C>T	Gln248*
	Glycosyltransferase family 4 protein	*orfN*	148dupG	Val50fs
	Cell division protein FtsL	*ftsL*	176G>A	Gly59Asp
	Transcriptional regulator	*lrp*	103G>T	Glu35*

* The amino acid led to a stop codon

** When hypermutators emerged, only those genetic events that may be involved in β-lactam resistance were included to simplify the analysis.

Supplementary Table 5. Genetic variations previously reported in clinical *P. aeruginosa* isolates or in experimental *in vitro* studies and leading to antibiotic resistance.

Gene (Amino acid modification)	Population	Studies	References
mexY (His908Leu)	TOB SCFM 3	Clinical studies	(1)
fusA (Thr671Ala)	TOB RM 1<br>TOB SCFM 1<br>TOB SCFM 2<br>TOB U 1<br>TOB U 2<br>TOB U 4		(1, 2)
fusA (Gln678Leu)	TOB SCFM 3		(1)
fusA (Arg680Cys)	TOB RM 4<br>TOB SCFM4		(1, 3)
fusA (Thr456Ala)	TOB U 3		(1)
ampC (Val239Gly)	CAZ SCFM 1		(1, 4)
anmK (Gly232Asp)	CAZ U 1		(1)
mpl (Met38fs)	CAZ U 1<br>CAZ U 2<br>CAZ-AVI SCFM 1<br>CAZ-AVI SCFM 2		(1, 4)

	CAZ-AVI SCFM 3<br>CAZ-AVI U 1<br>CAZ-AVI U 3		
mpl (Val384Gly)	CAZ SCFM 1<br>CAZ SCFM 2		(1)
mpl (Val124Gly)	CAZ RM 2		(4)
mpl (Tyr35Ser)	CAZ-AVI SCFM 3		(1)
dacB (Gln372*)	CAZ SCFM 4		(1)
dacB (Trp350Arg)	CAZ-AVI SCFM 1		(4)
ftsI (Arg504His)	CAZ SCFM 1<br>CAZ-AVI RM 1<br>CAZ-AVI SCFM 4		(1, 5, 6)
ftsI (Ala482Val)	CAZ SCFM 4		(7)
ftsL (Gly59Asp)	CAZ U 3<br>CAZ U 4<br>CAZ-AVI U 2<br>CAZ-AVI U 4		(1)
fusA (Thr671Ala)	TOB RM 1,<br>TOB SCFM 1,	Experimental studies	(8)

	TOB SCFM 2,<br>TOB U 1,<br>TOB U 2,<br>TOB U 4		
fusA (Gln678Leu)	TOB SCFM 3		(8)
fusA (Arg680Cys)	TOB RM 4,<br>TOB SCFM4		(8)
fusA (Thr456Ala)	TOB U 3		(8)
ftsI (Arg504His)	CAZ SCFM 1,<br>CAZ-AVI RM 1,<br>CAZ-AVI SCFM 4		(9)
orfN (Val50fs)	TOB RM 1,<br>TOB RM 2,<br>TOB RM 3,<br>TOB RM 4,<br>TOB U 3,<br>CAZ RM 2,<br>CAZ U 1,<br>CAZ U 2,<br>CAZ-AVI SCFM 1,<br>CAZ-AVI SCFM 2,<br>CAZ-AVI U 4		(8, 10, 11)

Supplementary Table 6. Genes reported to be mutated in clinical *P. aeruginosa* isolates or in experimental *in vitro* studies although the genetic variations are different.

Gene	Population	Studies	References
mexR	CAZ RM 1,<br>CAZ RM 3	Clinical studies	(4, 7, 12)
clpA	CAZ RM 1,<br>CAZ-AVI RM 1		(7)
nalD	CAZ RM 4,<br>CAZ-AVI RM 2,<br>CAZ-AVI RM 4,		(7, 12)
gyrB	CAZ SCFM 2		(13)
ampR	CAZ-AVI SCFM 2		(14)
mexB	CAZ RM 1,<br>CAZ RM 3,<br>CAZ-AVI RM 2,<br>CAZ-AVI RM 4		(4, 15)
mutL	CAZ-AVI U 1,<br>CAZ-AVI U 3		(4)
mutS	CAZ-AVI SCFM 1,		(4)

	CAZ-AVI SCFM 2		
mexT	TOB U 3		(4)
pmrB	TOB RM 1,<br>TOB RM 2,<br>TOB RM 3,<br>TOB RM 4		(4)
ptsP	TOB RM 2,<br>TOB SCFM 3,<br>TOB SCFM 4	Experimental studies	(8)
pmrB	TOB RM 1,<br>TOB RM 2,<br>TOB RM 3,<br>TOB RM 4		(8, 16)
orfH	TOB SCFM 1,<br>TOBSCFM 2,<br>TOB SCFM 4,<br>TOB U 3,<br>CAZ-AVI U 1,<br>CAZ-AVI U 2,<br>CAZ-AVI U 3		(8)
orfK	TOB SCFM 3,		(8)

	TOB U 1,<br>TOB U 2,<br>TOB U 4,		
mexR	CAZ RM 1,<br>CAZ RM 3		(7)
clpA	CAZ RM 1,<br>CAZ-AVI RM 1		(7)
ampC	CAZ SCFM 1,<br>CAZ-AVI SCFM 1,<br>CAZ-AVI SCFM 2,<br>CAZ-AVI SCFM 3		(7)
nalD	CAZ RM 4,<br>CAZ-AVI RM 2,<br>CAZ-AVI RM 4		(7)
pepA	CAZ-AVI RM 2		(7)
dacB	CAZ RM 4,<br>CAZ SCFM 1,<br>CAZ SCFM 2,<br>CAZ SCFM 3,		(7, 9)

	CAZ SCFM 4,<br>CAZ-AVI SCFM 1		
nalC	CAZ-AVI U 1,<br>CAZ-AVI U 3		(7)

Supplementary Table 7. Primers used to verify genetic modifications of the evolved populations in selected representative clones.

Gene	Genetic modification	Primer Fw (5’-3’)	Primer Rv (5’-3’)
fusA	2038C>T	CGGTAAGGTTATTCGTGCCG	TTTTAACCAGCGCTTCGACG
fusA	2011A>G		
fusA	1634G>A	GGACGAGAAGGGCAACATCA	CATGATCGGCTCGAGCACCT
ptsP	1135dupG	CTACCCGGAGCAGACCATC	TAGCCATCGACGATCAGGTC
ptsP	2156delG	TGCTGCATGCGTTGAAGAAG	AGCGAGCTGTGGATGACCTG
pmrB	853G>C	GCCGAACGCCGACTGACCAG	AATTGCTCCAGCAGGGCGTC
orfN	148delG	ATGGACGTTCCCAATGCCCG	CCGCCAGAATCAGCAAAACC
orfH	286C>T	CATGCGGCTGTGACTAATGG	GAATTACCAAATCGCCCGCT
orfH	561_565delGGCAGinsAGCTT	CCAGAGTCACGGAGATGCTT	TAGCTTGCACATTTCAGCGG
orfH	792delG	AGCTGATCCGACTCGCTAAT	ATTCGCTTGCAGAAACTCCG
orfK	355G>A	CTTGTCCATGGCGATACAGC	GGAGCAAAGTGAAGACAGGC
nuoD	183_184insC	TTACAAGGCAGACGACCAGG	GTAGAACACGCTGAAGTCGG
anmK	197G>A	GAAGACATCCTCGCCCTGT	TTTGACCGTGACTGCCGATA

mpl	111delC	CATCTGCGGGACCTTCATG	TTGCCGATCACTACCAGGTC
mpl	104A>C		
mpl	706A>C	CGAGGGCCTGATCATTCGC	GTTGTGCAGGCCGGTCAG
mpl	742C>T		
mpl	353C>G	GACCACGTGCTGCAAGGA	CGAAACCCCGAAGTTCTGC
mpl	416T>G	GAGCATGTCCTGAACAAGGG	CGGTAATGGACGAACTTCGA
ftsI	1510C>T		
ftsI	1511C>T	ATGCTGCAACAAGTGGTCG	CCGAAGTAGCCCGCCTTG
ftsI	1567C>T		
ftsL	176G>A	CGGCAGCTTCCTCATGTTG	CATGCGCAACTGTTCCACC
dacB	326G>A	ACCTATGCCGCCCTGGAAA	CGTCGTTGAATACCGGCAA
dacB	343C>T		
clpS	248A>C	GGTGGTACTGTTCAACGACG	CCCGTGCATACTGATTGACC
clpA	1634A>G	TCGTTCCTCTTCGCCGGT	CGTTGTTGTCGGTCAGGGT
dnaJ	1081G>A	AAGCTGTTCCGCCTGCGCG	ATAAAACCACTCCACGCG

PA14_45890	1001G>A	GCCAACGACCAGTTGTTCAA	ACCGTAGCGACGATCAGGGT
Del 299648 bp	Del 3200274-3499932	GCTTCACCGGTTCGCTGAAG<br>CTGTTCGAGATCGTCGAAGG	TCAGTCAATCGCCGCCCG<br>TTCGAACTCTTTCTCCTGTA
Del 220701 bp	Del 3288650-3509351	ATGCAACACAGCCAAGTGT<br>CTGTTCGAGATCGTCGAAGG	TCAGAACGGTTTGGTCGGCA<br>TTCGAACTCTTTCTCCTGTA
nalD	32G>T	AATACTTCGAGTCCGCCC	TTGAGCATCTCGTTGAACAG
pitA	367A>C	ATGTTCGATCTTTTCAGCGG	CGCGATGTCGATCGCCTT

REFERENCES

1. Hornischer K, Khaledi A, Pohl S, Schniederjans M, Pezoldt L, Casilag F, et al. BACTOME-a reference database to explore the sequence- and gene expression-variation landscape of Pseudomonas aeruginosa clinical isolates. Nucleic acids research. 2019;47(D1):D716-D20.
2. Bolard A, Plesiat P, Jeannot K. Mutations in Gene fusA1 as a Novel Mechanism of Aminoglycoside Resistance in Clinical Strains of Pseudomonas aeruginosa. Antimicrob Agents Chemother. 2018;62(2).
3. Chung JC, Becq J, Fraser L, Schulz-Trieglaff O, Bond NJ, Foweraker J, et al. Genomic variation among contemporary Pseudomonas aeruginosa isolates from chronically infected cystic fibrosis patients. J Bacteriol. 2012;194(18):4857-66.
4. Lopez-Causape C, Sommer LM, Cabot G, Rubio R, Ocampo-Sosa AA, Johansen HK, et al. Evolution of the Pseudomonas aeruginosa mutational resistome in an international Cystic Fibrosis clone. Sci Rep. 2017;7(1):5555.
5. Cabot G, Lopez-Causape C, Ocampo-Sosa AA, Sommer LM, Dominguez MA, Zamorano L, et al. Deciphering the Resistome of the Widespread Pseudomonas aeruginosa Sequence Type 175 International High-Risk Clone through Whole-Genome Sequencing. Antimicrob Agents Chemother. 2016;60(12):7415-23.
6. Kos VN, Deraspe M, McLaughlin RE, Whiteaker JD, Roy PH, Alm RA, et al. The resistome of Pseudomonas aeruginosa in relationship to phenotypic susceptibility. Antimicrob Agents Chemother. 2015;59(1):427-36.
7. Jorth P, McLean K, Ratjen A, Secor PR, Bautista GE, Ravishankar S, et al. Evolved Aztreonam Resistance Is Multifactorial and Can Produce Hypervirulence in Pseudomonas aeruginosa. mBio. 2017;8(5).
8. Scribner MR, Santos-Lopez A, Marshall CW, Deitrick C, Cooper VS. Parallel Evolution of Tobramycin Resistance across Species and Environments. mBio. 2020;11(3).
9. Cabot G, Zamorano L, Moya B, Juan C, Navas A, Blazquez J, et al. Evolution of Pseudomonas aeruginosa Antimicrobial Resistance and Fitness under Low and High Mutation Rates. Antimicrob Agents Chemother. 2016;60(3):1767-78.
10. Hernando-Amado S, Sanz-García F, Martínez JL. Antibiotic Resistance Evolution Is Contingent on the Quorum-Sensing Response in Pseudomonas aeruginosa. Mol Biol Evol. 2019;36(10):2238-51.
11. Sanz-Garcia F, Sanchez MB, Hernando-Amado S, Martinez JL. Evolutionary landscapes of Pseudomonas aeruginosa towards ribosome-targeting antibiotic resistance depend on selection strength. Int J Antimicrob Agents. 2020;55(6):105965.
12. Sobel ML, Hocquet D, Cao L, Plesiat P, Poole K. Mutations in PA3574 (nalD) lead to increased MexAB-OprM expression and multidrug resistance in laboratory and clinical isolates of Pseudomonas aeruginosa. Antimicrob Agents Chemother. 2005;49(5):1782-6.
13. Pasca MR, Dalla Valle C, De Jesus Lopes Ribeiro AL, Buroni S, Papaleo MC, Bazzini S, et al. Evaluation of fluoroquinolone resistance mechanisms in *Pseudomonas aeruginosa* multidrug resistance clinical isolates. Microb Drug Resist. 2012;18(1):23-32.
14. Cabot G, Ocampo-Sosa AA, Dominguez MA, Gago JF, Juan C, Tubau F, et al. Genetic markers of widespread extensively drug-resistant Pseudomonas aeruginosa high-risk clones. Antimicrob Agents Chemother. 2012;56(12):6349-57.
15. Marvig RL, Sommer LM, Molin S, Johansen HK. Convergent evolution and adaptation of Pseudomonas aeruginosa within patients with cystic fibrosis. Nat Genet. 2015;47(1):57-64.
16. Lopez-Causape C, Rubio R, Cabot G, Oliver A. Evolution of the Pseudomonas aeruginosa Aminoglycoside Mutational Resistome In Vitro and in the Cystic Fibrosis Setting. Antimicrob Agents Chemother. 2018;62(4).
